# Glomerulotubular pathology in dogs with subclinical ehrlichiosis

**DOI:** 10.1371/journal.pone.0260702

**Published:** 2021-12-08

**Authors:** Leandro Zuccolotto Crivellenti, Cristiane Alves Cintra, Suellen Rodrigues Maia, Gyl Eanes Barros Silva, Sofia Borin-Crivellenti, Rachel Cianciolo, Christopher A. Adin, Mirela Tinucci-Costa, Caio Santos Pennacchi, Aureo Evangelista Santana

**Affiliations:** 1 Graduate Program in Veterinary Science (PPGCV)/College of Veterinary Medicine (FAMEV), Universidade Federal de Uberlândia (UFU), Uberlândia, Brazil; 2 Department of Veterinary Clinic and Surgery, Faculdade de Ciências Agrária e Veterinária (FCAV), Universidade Estadual Paulista Júlio de Mesquita Filho (UNESP), Jaboticabal, Brazil; 3 Department of Veterinary Clinics, Faculdade de Medicina Veterinária e Zootecnia (FMVZ), Universidade Estadual Paulista Júlio de Mesquita Filho (UNESP), Botucatu, Brazil; 4 Department of Pathology, Universidade Federal do Maranhão (UFMA), São Luís, Brazil; 5 Department of Veterinary Biosciences, College of Veterinary Medicine, The Ohio State University, Columbus, Ohio, United States of America; 6 Department of Small Animal Clinical Sciences, University of Florida, Gainesville, Florida, United States of America; Kansas State University, UNITED STATES

## Abstract

Subclinical stage of ehrlichiosis is characterized by absence of clinical or laboratory alterations; however, it could lead to silent glomerular/tubular changes and contribute significantly to renal failure in humans and animals. The aim of this study was to evaluate glomerular and tubular alterations in dogs with subclinical ehrlichiosis. We evaluated renal biopsies of 14 bitches with subclinical ehrlichiosis and 11 control dogs. Samples were obtained from the left kidney, and the tissue obtained was divided for light microscopy, immunofluorescence, and transmission electron microscopy. Abnormalities were identified by light microscopy in 92.9% of dogs with ehrlichiosis, but not in any of the dogs of the control group. Mesangial cell proliferation and synechiae (46.1%) were the most common findings, but focal segmental glomerulosclerosis and ischemic glomeruli (38.4%), focal glomerular mesangial matrix expansion (30.7%), mild to moderate interstitial fibrosis and tubular atrophy (23%), and glomerular basement membrane spikes (23%) were also frequent in dogs with ehrlichiosis. All animals with ehrlichiosis exhibited positive immunofluorescence staining for immunoglobulins. Transmission electron microscopy from dogs with ehrlichiosis revealed slight changes such as sparse surface projections and basement membrane double contour. The subclinical phase of ehrlichiosis poses a higher risk of development of kidney damage due to the deposition of immune complexes.

## Introduction

Canine monocytic ehrlichiosis (CME), caused by the bacteria *Ehrlichia canis*, has an incubation period of eight to twenty days [1]. This period is followed by the acute, subclinical, and chronic phases, which are classified according to clinical signs and clinicopathological abnormalities [2, 3]. Although clinical signs of naturally infected animals can manifest differently from those found in experimental infections [4, 5], the acute phase is easily recognized due to the presence of clinical signs [2, 3, 6, 7]. It is known that, in this phase, proteinuria [2, 3] and minimal change diseases [8] or amyloidosis [9] can occur.

Following the acute phase of the disease, the subclinical phase has a variable duration, which can extend from months to years [10]. *Ehrlichia canis* infection may persist after spontaneous clinical recovery or ineffective treatment, and infected animals may enter the subclinical stage of CME [11].

In the subclinical stage, animals exhibit no clinical signs and can manifest mild laboratory abnormalities [11, 12]. This phase is marked by high and persistent antibody titers in serum due to trapping of bacteria in the tissues [13]. It is probable that this constant stimulation of the immune system may cause glomerulonephritis induced by deposition of immune complexes [8, 14]. In addition, chronic immune system stimulation caused by *Ehrlichia sp*. would lead to a silent glomerular/tubular alteration and contribute significantly to renal failure in humans [15, 16] and animals [17–19]. This condition is reinforced by the evidence that dogs infected with *Ehrlichia sp*. are at increased risk of developing CKD [20]; however, no renal histopathological analysis has been done to confirm this hypothesis.

The aim of this study was to evaluate the glomerular and tubular alterations of dogs with subclinical ehrlichiosis.

## Materials and methods

The following study was approved by the Veterinary Ethics Committee of UNIFRAN–Universidade de Franca (protocol no. 069/15).

A prospective study was performed in intact female dogs presented for surgical sterilization (elective) at the Veterinary Teaching Hospitals of UNIFRAN and Universidade Estadual Paulista (UNESP). After obtainment of permission from the tutors, 25 intact dogs from an endemic area for ehrlichiosis, with no clinical signs of the disease, no physical alterations, nor any changes in pre-surgical exams (complete blood count (CBC), biochemical profile and blood pressure) were selected for the experiment. Animals with urine with macroscopic hematuria were not included. Dogs with neoplasms, azotemia, or concurrent endocrinopathies were excluded from the study.

The control (control) and subclinical *Ehrlichia* (ehrlichiosis) groups were selected based on negative and positive results for *E*. *canis*, respectively. Both results were evaluated by polymerase chain reaction (PCR) and serology (Immunocomb^®^ - Biogal, Israel.commercial kit) (sensitivities 0.86 and specificities 0.98) [21, 22].

The PCR involved the 16S rRNA gene from *E*. *canis* and the primers initially used were the ECC primer (59-AGAACGAACGCTGGCGGCAAGCC-39) and the ECB primer (59-CGTATTACCGCGGCTGCTGGC-39), followed by HE-3 primer (59-TATAGGTACCGTCATTATCTTCCCT) and ECA primer (59-CAATTATTTATAGCCTCTGGCTATAGGAA-39).

In addition, animals from both groups were was also negative for *Anaplasma* sp. and *Babesia* sp. as assessed by PCR, and negative for leptospirosis (L. icterohaemorrhagiae, L. canicola, L. pomona and L. grippotyphosa) as evaluated by dot-ELISA point-of-care (Test-ITTM Leptospira canina IgM and IgG—Biogal, Israel.com commercial kit).

The control group consisted of 11 bitches with an average age of 5.8 ± 3.4 years and an average weight of 12.8 ± 17.2 kg. The ehrlichiosis group consisted of 14 bitches with an average age of 5.3 ± 2.5 years and an average weight of 14.7 ± 9.2 kg; these animals were clinically asymptomatic, but positive for *E*. *canis* as described above (titers greater than 1:320) (Table 1).

**Table 1:** Demographic, physical characteristics, and molecular and serological findings of the dogs selected in the study.

	GROUPS	
	Control (n = 11)	Ehrlichiosis (n = 14)
**Age (years)**	5.8 ± 3.4	5.3 ± 2.5
**Weight (kg)**	12.8 ± 17.2	14.7 ± 9.2
**Sex**	female	female
**Coming from an endemic area for ehrlichiosis?**	Yes	Yes
**PCR**		
*Ehrlichia* sp.	Negative	Positive
*Anaplasma* sp.	Negative	Negative
*Babesia* sp.	Negative	Negative
**Serology**		
*Ehrlichia canis*	Negative	Positive (titers >1:320)
Leptospirosis	Negative	Negative

Urine and blood samples were collected 1 hour before ovariohysterectomy (control group or ehrlichiosis group). Complete blood count (CBC), biochemical profile (creatinine, urea, alanine aminotransferase [ALT], alkaline phosphatase [ALP], phosphorus, calcium, albumin, total protein, sodium, and potassium), and blood pressure values (determined by Doppler ultrasound) were obtained in order to assess possible abnormalities that would exclude the animals from the control group, as well as to evaluate the possibility of pre- or post-renal causes of possible proteinuria in the animals of the ehrlichiosis group. Urinalysis and urine protein/creatinine ratio (UPC) (samples obtained by cystocentesis) were also performed for each dog.

Open renal biopsy was performed in all dogs at the time of ovariohysterectomy. Samples were obtained from the left kidney using an 18, 16 or 14 gauge (G) semi-automated cutting needle (M.D.L Aghi specialli e componenti medicalli—Italy) and the biopsied tissue was divided for LM, IF, and TEM.

Bouin-fixed samples were used for LM, and sections were cut at 2–3 μm thickness. Four different stains were used to evaluate the kidney samples: hematoxylin and eosin (HE), periodic acid Schiff (PAS) (for the morphometric analyzes of the different glomerular components and the analysis of glomerulosclerosis), Jones methenamine silver (JMS), and Masson’s trichrome (TRI) (for the analysis of glomerular collagen). When amyloid deposition was suspected, Congo Red staining was also performed on sections of 10 μm thickness. Two samples were analyzed by LM (> 10 glomeruli).

For direct IF, Michel’s solution was used to transport the sample. Fresh unfixed renal specimens were washed with PBS, and then were embedded in optimal cutting temperature (OCT) compound. Samples were then snap-frozen in liquid nitrogen. Subsequently, 3–4 μm-thick sections were cryosectioned and fixed with acetone for 15 minutes. After washing twice with PBS, the slides were incubated with polyclonal FITC-labeled goat anti-dog IgA, IgG, IgM, and complement C3 antibodies (Bethyl Laboratories). Immunofluorescence examination was classified as granular or linear, and included the location of the deposit (mesangium, capillary walls, tubules, or blood vessels), distribution (focal, diffuse, segmental or global), and intensity. Immunofluorescence staining intensity was assessed using a semi-quantitative scale, ranging from 0 to 3+ (0 negative, 1+ weak staining, 2+ moderate staining, 3+ strong staining).

Samples for electron microscopy were fixed using 2.5% glutaric dialdehyde solution and kept refrigerated. Subsequently, they were post-fixed in 0.2M sodium cacodylate buffer and 1% osmium tetroxide, and then dehydrated through a series of acetone solutions. After that, the material was embedded in Araldite resin and sectioned into ultrathin slices.

All paraffin and frozen sections were examined by a physician nephropathologist who was blinded to the ehrlichiosis status of the animals. Renal histological diagnoses were classified according to the World Health Organization’s classifications of human glomerular diseases as used in other studies [23–26].

Descriptive statistics and frequency distribution of variables were evaluated in each group. Normally distributed data were tested using students’ t-test. Immunofluorescence score data was not normally distributed and statistical comparisons were performed using Kruskal-Wallis analysis, followed by the Dunns’ post-hoc test. The normality of the values was assessed using Kolmogorov-Smirnov and Shapiro-Wilk tests. Significance was set at p<0.05. Associations between histological alterations (subjectively graded) and urinary and serum parameters were evaluated using Pearson’s correlation. Calculations were performed using GraphPad Prism 7.0 software.

## Results

That as expected the all dogs with ehrlichiosis had high antibody titers on the "Dot-ELISA" test for detection of IgG anti-*Ehrlichia canis* (i.e. >1:320, supporting evidence of subclinical infection) [21, 22].

The parameters studied are specified in Table 2. CBC showed no difference between the groups, but five (35.7%) and four animals (28.57%) animals in the ehrlichiosis group had erythrocyte and platelet concentrations at the lower limit, respectively.

**Table 2:** Biochemical, urinary specific gravity, urine protein/creatinine ratio (UPC), systolic blood pressure (SBP) and hematological parameters (mean ± std deviation) of dogs from the control (n = 11) and ehrlichiosis group (n = 14).

	Reference intervals	GROUPS		*P value*
		Control	Ehrlichiosis	
RBC count (x10^6^/μL)	5,5–8,4	6.37 ± 0.69	5.85 ± 1.58	0.9351
Hemoglobin (g/dL)	12–18	14.2 ± 1.7	13.3 ± 3.4	0.9701
HCT (%)	37–55	42 ± 4	39 ± 11	0.9658
Leukocytes (x10^3^/μL)	6–18	8,782 ± 1,871	9,793± 5,769	0.4752
Platelets (x10^3^/μL)	180–480	316.5 ± 72.3	283.2 ± 115.9	0.9053
Total protein (g/L)*	5.4–7.1	5.9 ± 0.6	7.3 ± 0.9	0.0007
Albumin (g/L)*	2.4–4.0	3.1 ± 0.4	2.7 ± 0.6	0.0242
Globulin (g/L)*	2.7–4.4	2.8 ± 0.7	4.3 ± 1.2	0.0039
Creatinine (mg/dL)	0.5–1.4	1.0 ± 0.2	0.9 ± 0.1	0.0982
Urea (mg/dL)	15–65	27.7 ± 11.6	34.9 ± 15.1	0.2204
Urinary Specific Gravity *	1.020–1.045	1.039 ± 0.01	1.032 ± 0.009	0.0319
UPC*	< 0.2 (Non-proteinuric)	0.12±0.07	0.45±0.57	0.0215
	0.2–0.5 (Borderline proteinuric)			
	> 0,5 (Proteinuric)			
SBP (mmHg)	< 140.0	125.0 ± 13.85	132.2 ± 12.28	0.4375
Findings glomerulotubular alterations	LM	Normal findings	Abnormalities in 92.9%	
	IF	Negative	Positive in 100%	
	TEM	Normal findings	Abnormalities in 55.6%	

(*) Variables that show statistical differences.

In the biochemical profile, the animals with ehrlichiosis had significant increases in the following parameters compared to the control group: total protein (5.94 ± 0.62 versus 7.31 ± 0.88; P <0.0001) and globulins (2.81 ± 0.68 versus 4.32 ± 1.24, P <0.0001), and significantly decreased albumin levels (3.09 ± 0.37 versus 2.74 ± 0.56, P = 0.0187). Systolic blood pressure did not vary significantly between groups, and remained within normal limits for the species.

The ehrlichiosis group had lower urinary specific gravity compared to the control group, however, both values were hyperstenuric and within the reference range.

### Proteinuria

The urinary protein creatinine ratio (UPC) was significantly higher in dogs of the ehrlichiosis group compared to the control group (0.45±0.57 versus 0.12±0.07, P<0.0215). No control animals presented proteinuria, whereas 28.6% (n = 4) of the dogs of the ehrlichiosis group presented values of UPC> 0.5 (Table 1). None of the dogs exhibited protein above 2.0 in the urine sampled prior to surgery. None of the animals had active urinary sediment. Red blood cells were found in moderate numbers (10–20 RBC/hpf) in 3 dogs from the control group and in 2 from the ehrlichiosis group, (5–7 RBC/hpf) in 3 of the control and 7 of the ehrlichiosis group, and no red blood cells in the sediment of 5 of the control and 5 of the ehrlichiosis group.

In addition, there were moderate positive correlations between UPC and serum globulin concentrations (P = 0.035; r = 0.44) and immunofluorescence deposition (P = 0.022, r = 0.466), and a moderate negative correlation with serum albumin (P = 0.036, r = - 0.439).

### Light microscopy

**Fig 1:**
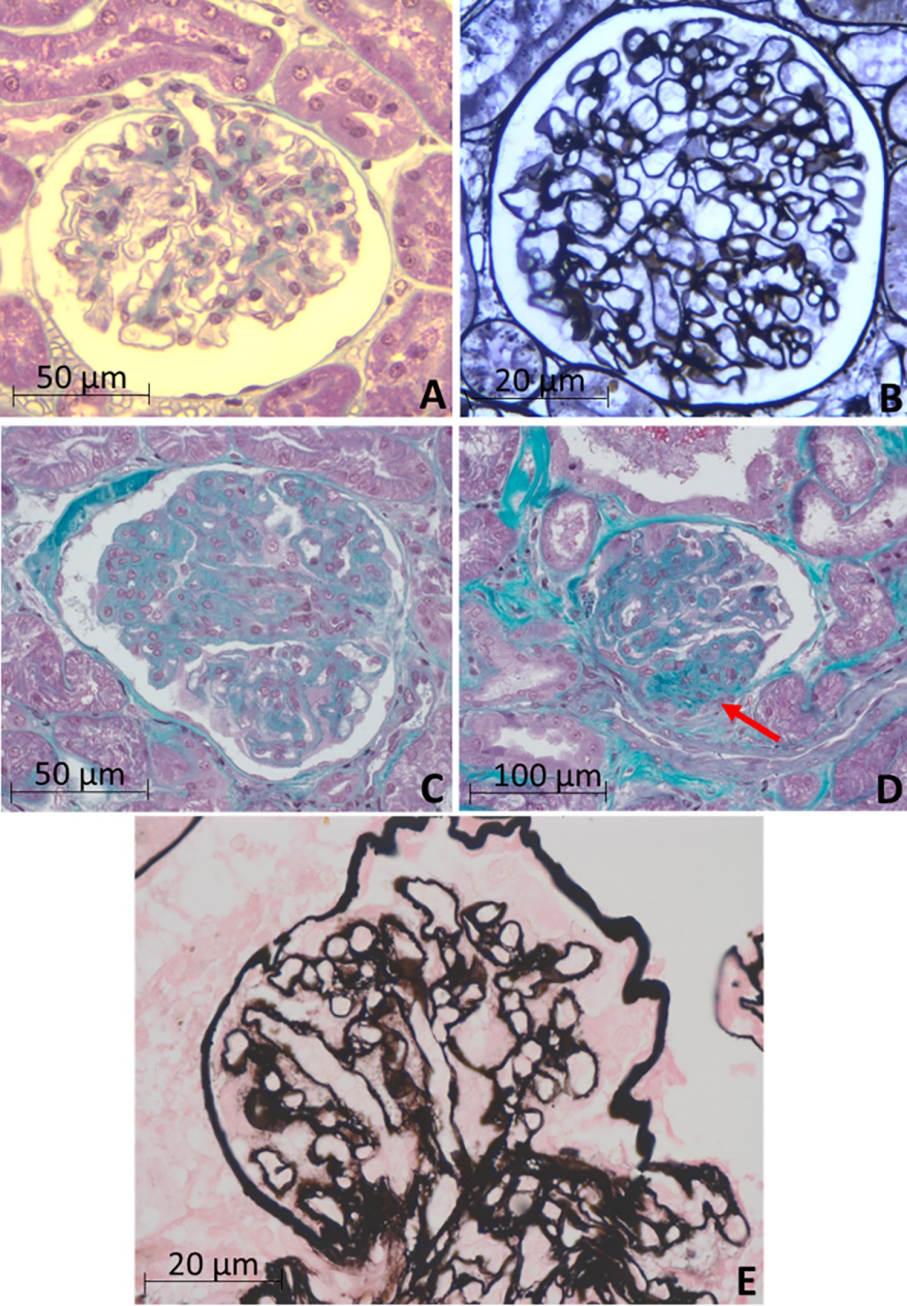
Light microscopy findings. Control group: (A) Glomerular structure preserved, with no evidence of alterations. Masson’s Trichrome (x400). (B) Preserved glomerular structure, without evidence of alterations. Jones methenamine silver (JMS) (x630). Ehrlichiosis group: (C) Membranous-like glomerulopathy with diffuse and regular thickening of the glomerular capillary walls, frequently with mild and segmental mesangial proliferation. Masson’s trichrome (x400). (D) Synechia with evident segmental glomerulosclerosis (arrow). Masson’s Trichrome (x400). (E) Membranous-like glomerulopathy with sparse surface projections (spikes) along sub-epithelial surface of the glomerular basement membrane (arrow) (JMS) (x630).

There were no changes in light microscopy of animals in the control group (Fig 1A and 1B), whereas light microscopy yielded abnormal results in 92.9% of dogs with ehrlichiosis (n = 13). The most common glomeruli changes were mesangial proliferation (n = 6, 46.1%) (Fig 1C) and synechiae (adhesion of the glomerular membrane to the capsule) (n = 6, 46.1%) with evident segmental glomerulosclerosis (n = 5, 38.4%) (Fig 1D), ischemic glomeruli (n = 5, 38.4%), thickening of the glomerular basement membrane (n = 4, 30.7%), and silver stain showed sparse surface projections (spikes) along the subepithelial surface of the glomerular basement membrane (n = 3, 23%) (Fig 1E). Tubule-interstitial evaluation showed hydropic degeneration of tubular epithelial cells (n = 4, 30.7%), mild to moderate interstitial fibrosis and tubular atrophy (IFTA) (n = 3, 23%), and tubular hyaline droplets (n = 3, 23%). Two animals (15.3%) presented hyaline arteriolosclerosis. No changes were observed by light microscopy in the control group. A moderate positive correlation of light microscopy with globulin concentration (P <0.001, r = 0.669) was observed.

### Immunofluorescence

**Fig 2:**
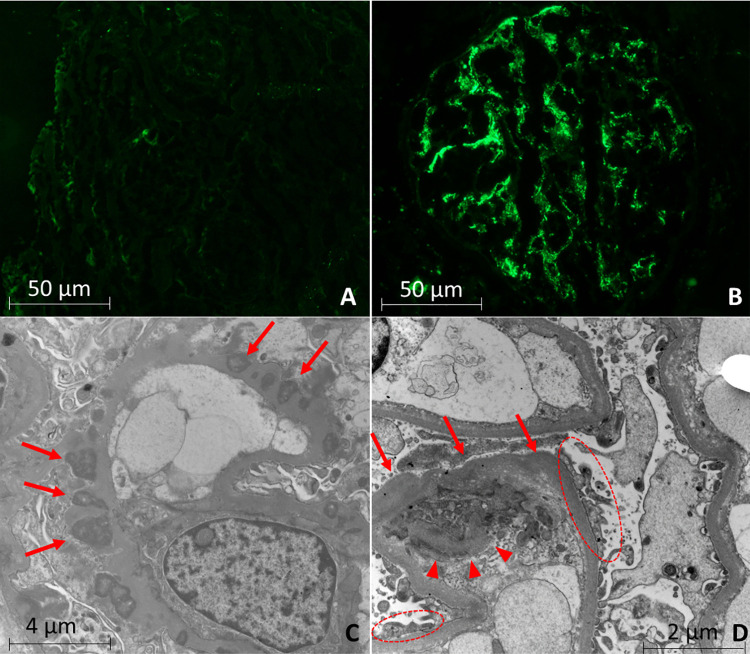
Immunofluorescence and transmission electron microscopy findings. Control group: (A) Negative immunofluorescence. (x400). Ehrlichiosis Group: (B) Strong diffuse granular positivity for IgM in mesangium. (x400). (C) Electron microscopy evidencing sub-epithelial (arrow) and (D) sub-endothelial deposits (arrow) and capillary duplication of glomerular basement membrane (arrowhead) and effacement of the podocyte foot processes (dashed circle).

All animals with ehrlichiosis exhibited positive immunofluorescence staining for immunoglobulins. Of these, nine stained only for IgM (64.3%), two for IgM/IgA (14.3%), one for IgM/C3 (7.1%), one for IgM/IgA/C3 (7.1%) and one for IgM/IgG/IgA/C3 (7.1%). Immunoglobulins were predominantly distributed in a granular pattern in the loop and in the mesangium (Fig 2B). Moderate-intensity immunofluorescence was observed for IgG and IgA in the mesangium and glomerular capillaries of one animal with ehrlichiosis. C3 staining was observed in three animals (21.4%) (strong in one animal and weak in the others). A positive result for IgM was seen as strong, moderate, and weak in 42.8%, 35.7% and 21.4%, respectively. The intensity of immunofluorescence was weak for IgA in three animals (75%) and moderate in one (25%). For all IgG positive animals, the verified intensity was weak (Table 3). The control group did not present immunostaining, which is considered to be negative (Fig 2A) (S1 Table).

**Table 3:** Relative distribution of the immunofluorescence intensity score in ehrlichiosis group. The same animal may have been positive for more than one protein.

Immunofluorescence Intensity Score	Ehrlichiosis group (n = 14)			
	IgG (n = 1)	IgA (n = 4)	IgM (n = 14)	C3 (n = 3)
Weak	100%	75%	21.4%	66.6%
Moderate		25%	35.7%	
Strong			42.8%	33.3%

### Transmission electron microscopy

From the 25 kidney biopsies, 5 dogs with ehrlichiosis had no glomeruli on the samples for TEM evaluation. Transmission electron microscopy of renal biopsies from dogs of the ehrlichiosis group revealed slight subepithelial and mesangial electron-dense deposits (n = 5, 55.6%), as well as projection spikes and capillary loops duplication of the glomerular basement membrane (n = 3, 33.3%) (Fig 2C), with mesangial deposits consistent with immune complexes. Segmental effacement of podocyte foot processes was identified in seven of the animals with *E*. *canis* (77.8%). Two animals (22.2%) had subendothelial deposits of electron-dense material (Fig 2D). It was possible to observe the presence of glomerular hematuria in one of the animals. The control group did not exhibit alterations by electron microscopy examination.

## Discussion

The results of our study provide strong evidence that dogs naturally infected with *Ehrlichia canis* in the subclinical phase are at high risk of developing kidney damage. It is important to note that all dogs included in this study were selected without clinical and laboratory evidence of any disease, including visiting the hospital only for elective surgical treatment (sterilization), which further reinforces our findings.

The increased serum globulin concentrations observed in this study may be due to a humoral response, which would occur as a consequence of the antigenic stimulus promoted by the persistence of Ehrlichia in the body [8, 27], as seen by the strong seropositivity. Thus, as described in the literature [8, 14], this antigenic stimulation, resulting from the humoral response in numerous disease conditions (infectious and noninfectious), is a known mechanism that induces glomerulopathy and consequent proteinuria, reinforcing our results: hyperglobulinemia, renal injury and proteinuria in the group of infected animals.

Although the ehrlichiosis group presented values significantly higher than the control group, the mean UPC value was 0.4, considered borderline, and also without clinical significance in non-azotemic patients according to International Renal Interest Society [28]. Since even patients with borderline UPC may present renal damage, further testing should be considered, such as the evaluation of microalbuminuria [29] and/or urinary electrophoresis [30]. It also may be reinforced by the result coming from the recent retrospective study [31], which demonstrated that about 34% of proteinuric dogs were exposed to diseases transmitted by canine vectors. Another possibility would be the occurrence of renal hematuria, which although widely recognized in humans [32–34], is still very little investigated in veterinary medicine. A case of renal hematuria was observed by electron microscopy in the tubule and in the glomerular loops (n = 1, 7.69%) in this study, and although it may have been observed at random, this type of evaluation and finding could be the focus of future discussions.

The expansion of the mesangial glomerular matrix and mesangial proliferation, which were observed in this study, characteristics of membranoproliferative nephropathy, corroborate the descriptions in the literature [23, 35, 36], which characterize the main renal histopathological changes resulting from infectious diseases, among which, those transmitted by ticks [36, 37]. Furthermore, subepithelial lesions have also been found and are consistent with deposits of immune complexes, which is a characteristic histopathological feature of membranous nephropathy [38]. Such findings (membranoproliferative nephropathy and membranous nephropathy) can be described as forms of immune-mediated glomerulonephritis (IMGN) [36], and we believe they are related to the hyperstimulation of the immune system that occurs against *E*. *canis* in dogs in the subclinical and acute phases of the disease [39]. Although the pathogenesis of the disease is not yet fully elucidated, this immune response (stronger in the acute phase, but persistent in the subclinical phase) is marked by an increase in nitrite / nitrate and pro-inflammatory cytokines, among which TNF- α [40], while levels of anti-inflammatory cytokines (IL-10) are low in serum [39]. There is also an increase in immunoglobulins, CD3+ and CD8+ cells in different tissues of infected dogs, findings that reinforce both the role of the humoral response and the cellular response in the pathogenesis of the disease [7].

Advanced age and hypertension can lead to hyaline arteriolosclerosis [41], and even though this was observed in two dogs from the Ehrlichiosis group, the blood pressure obtained was lower than 150 mmHg and their ages were 3 and 8 years, suggesting that this abnormality in dogs with subclinical ehrlichiosis could be caused by some other disorder.

Despite the fact that platelet counts within the lower limits of the normal range in four animals, which is a characteristic of the subclinical phase of the disease [12, 42–44], no animal presented complications following the renal biopsy procedure, and macroscopic hematuria ceased within 48 hours in all cases, as described in the literature [23].

Passive trapping of immunoglobulin deposits may be present in the cases that exhibited positive staining for IgM, since IgM is a large glycoprotein (880 to 941 kD) and can get trapped nonspecifically in glomeruli [30], but deposition of immune complexes is a possibility, since the granular pattern has been associated with C3 deposition and this finding, together with the existence of positivity for immunoglobulins, reinforces the diagnosis of deposits of immune complexes [41].

Another evidence that the glomerulopathy may be caused by immune complexes is the observation of subepithelial and mesangial deposits with spike projections and capillary loops duplication of the glomerular basement membrane assessed by TEM [8, 23]. Segmental podocyte foot process effacement was observed as a sporadic and focal finding in the ehrlichiosis group, such as reported for animals in the acute phase [8]; however, these findings do not agree with minimal change glomerulopathy according to the current guidelines [41]. Such alterations may contribute to the increase of proteinuria, as seen in dogs with mammary neoplasia [30].

Dogs diagnosed with ehrlichiosis are known to have a 112% higher risk of developing CKD compared to animals that do not develop the infection [20]. Because of that, our results may hypothesize that kidney injuries suffered from subclinical stages may contribute to the chronicity of changes, impacting this type of future repercussion.

The results of our study provide evidence that dogs naturally infected with *Ehrlichia canis* in the subclinical stage have a higher risk of developing kidney damage, and this is related to the deposition of immune complexes.

## Supporting information

S1 Table Renal biopsy immunofluorescence in both groups. (DOCX)
