# Supplementary material for: Glomerulotubular pathology in dogs with subclinical ehrlichiosis
Source: PLoS One. 2021 Dec 8;16(12):e0260702. doi: 10.1371/journal.pone.0260702 (PMC8654155; doi:10.1371/journal.pone.0260702)
Supplement: S1 Table — (DOCX) [file pone.0260702.s001.docx]

**S1 Table.** Renal biopsy immunofluorescence in both groups.

| GRUPS | Immunofluorescence intensity score | | | |
| --- | --- | --- | --- | --- |
|  | **IgM** | **IgG** | **IgA** | **C3** |
| Ehrlichiosis | 2 | 1 | 1 | 1 |
| Ehrlichiosis | 2 | 0 | 0 | 0 |
| Ehrlichiosis | 1 | 0 | 0 | 3 |
| Ehrlichiosis | 1 | 0 | 0 | 0 |
| Ehrlichiosis | 3 | 0 | 0 | 0 |
| Ehrlichiosis | 3 | 0 | 0 | 0 |
| Ehrlichiosis | 1 | 0 | 0 | 0 |
| Ehrlichiosis | 2 | 0 | 1 | 0 |
| Ehrlichiosis | 2 | 0 | 0 | 0 |
| Ehrlichiosis | 3 | 0 | 2 | 1 |
| Ehrlichiosis | 3 | 0 | 0 | 0 |
| Ehrlichiosis | 3 | 0 | 0 | 0 |
| Ehrlichiosis | 2 | 0 | 1 | 0 |
| Ehrlichiosis | 3 | 0 | 0 | 0 |
| Control | 0 | 0 | 0 | 0 |
| Control | 0 | 0 | 0 | 0 |
| Control | 0 | 0 | 0 | 0 |
| Control | 0 | 0 | 0 | 0 |
| Control | 0 | 0 | 0 | 0 |
| Control | 0 | 0 | 0 | 0 |
| Control | 0 | 0 | 0 | 0 |
| Control | 0 | 0 | 0 | 0 |
| Control | 0 | 0 | 0 | 0 |
| Control | 0 | 0 | 0 | 0 |
| Control | 0 | 0 | 0 | 0 |

0 negative, 1+ weak staining, 2+ moderate staining, 3+ strong staining.
